# UAV Landing Based on the Optical Flow Videonavigation

**DOI:** 10.3390/s19061351

**Published:** 2019-03-18

**Authors:** Alexander Miller, Boris Miller, Alexey Popov, Karen Stepanyan

**Affiliations:** Institute for Information Transmission Problems RAS, Bolshoy Karetny per. 19, Build.1, Moscow 127051, Russia; amiller@iitp.ru (A.M.); ap@iitp.ru (A.P.); KVStepanyan@iitp.ru (K.S.)

**Keywords:** UAV, landing, optical flow, video navigation, Kalman filter

## Abstract

An automatic landing of an unmanned aerial vehicle (UAV) is a non-trivial task requiring a solution of a variety of technical and computational problems. The most important is the precise determination of altitude, especially at the final stage of approaching to the earth. With current altimeters, the magnitude of measurement errors at the final phase of the descent may be unacceptably high for constructing an algorithm for controlling the landing manoeuvre. Therefore, it is desirable to have an additional sensor, which makes possible to estimate the height above the surface of the runway. It is possible to estimate all linear and angular UAV velocities simultaneously with the help of so-called optical flow (OF), determined by the sequence of images recorded by an onboard camera, however in pixel scale. To transform them into the real metrical values it is necessary to know the current flight altitude and the camera angular position values. The critical feature of the OF is its susceptibility to the camera resolution and the shift rate of the observed scene. During the descent phase of flight, these parameters change at least one hundred times together with the altitude. Therefore, for reliable application of the OF one needs to coordinate the shooting parameters with the current altitude. However, in case of the altimeter fault presence, the altitude is also still to be estimated with the aid of the OF, so one needs to have another tool for the camera control. One of the possible and straightforward ways is the camera resolution change by pixels averaging in computer part which performed in coordination with theoretically estimated and measured OF velocity. The article presents results of such algorithms testing from real video sequences obtained in flights with different approaches to the runway with simultaneous recording of telemetry and video data.

## 1. Introduction

Today the development of optoelectronic devices and data transmission systems allows to use them in remote control systems of unmanned aerial vehicles (UAV). In case of remote control which is carried out by an operator the characteristics of the optical devices and the transmitted image must correspond to the capabilities of human vision. However, in case of the UAV autonomous flight, the optical systems and the onboard computer must work together solving the problems of identifying the observed objects with their coordinates. It means that requirements to characteristics of optoelectronic devices are different from remote flight control case. The UAV control system which includes an optoelectronic system (OES) and an onboard computer determines the movement of the onboard video camera and identifies the objects observed in the field of view of the camera [1]. There are several approaches to the usage of OES [2]. The first one is to detect and to track the movement of specific local areas (reference points) on the image by analogy with human vision [3,4]. With this approach, it is easy to transform the recorded images into metric values for the control system. Some examples of this approach to UAV navigation are as follows:

- the tracking of singular points and development of the algorithm based on determining of their angular coordinates and establishing of correspondence of their images in preliminary uploaded template map based on RANSAC methodology described in [5];
- conjugation of the rectilinear objects segments such as walls of buildings and roads are in [6];
- fitting of characteristic curvilinear elements [7];
- matching of textured and coloured areas [8,9];
- matching of epipolar lines, such as runways, at landing [10].

One possible reference to this approach is *sparse OF*. An example of this approach to the UAV landing in case of the smoke occlusions basing on the natural terrain landmarks is given in [11]. The second approach is based on non-metric analysis and is built by analogy with a vision of insects or birds [12]. Nowadays an approach that uses information containing in the vector field of the image motion (in other words in the optical flow (OF)) is developing. OF is well known to engineers developing cameras for shooting from moving carriers where unreduced OF leads to the resolution degradation and needs reduction either optomechanically [13,14] or electronically [15]. At the same time in the UAV application area, the OF contains the information about the carrier’s velocities and thereby may serve as an additional sensor for the UAV navigation.

OF is the projection of the camera’s motion onto the focal plane. OF generates the nonuniform field of image shifts velocities which is known as *dense optical flow*. In case of tracking the displacement of some reference points on the underlying surface, it is common to use the *sparse optical flow* term. The methodology of the OF computation had been developed long ago, mainly for estimation of quality for various optomechanical image shift compensation systems performance [16]. Nowadays there are various examples of the OF usage in UAV applications such as:

- in landing at the unknown hazardous environment with the choice of the landing place [17];
- in experimental landing with the aid of special landing pads [18];
- vision based and mapping for landing with the aid of model predictive control [19];
- in landing manoeuvring of the UAV [20,21];
- in tracking tasks of linear objects such as communication lines and pipelines on the terrain [22];
- even in usual manoeuvring [23];
- slope estimation for autonomous landing [24];
- in distance estimation with application to the UAV landing with the aid of the mono camera [25].

Our research team is working on video navigation as an additional navigation tool for UAV of aeroplane type. We consider OF as an additional tool for estimation of the UAV velocity in the absence of specific regions on the earth surface which can serve as beacons for estimation of the UAV position. The specific feature of the OF is that it gives information about velocities only but not on position. Therefore, the bias of the position estimates which inevitably exists at the beginning of the flight path can only increase without additional intermediate corrections. Correct filtering algorithm can reduce this bias but cannot eradicate it. It means that it is essential to evaluate the specific noises related to the OF estimation, so in our experiments, we perform flights where qualified pilot performs the series of approaches to the runway, during which one can carefully record the video sequences and corresponding telemetry data. These data serve as a source of information about specific OF noises. Filtering method fuses the data at UAV altitude estimation during landing.

In the existing literature, the OF usage at landing generally relates to the copter type UAV [11]. Here it is possible to coordinate velocity of descent with the observable video sequence. There are a series of works demonstrating successful approaches to the copter control based on the divergence observation. Here the divergence of the OF field serves as a measure of the approach velocity to the earth surface. Almost all articles were presenting the successful application of the OF relate to micro air vehicles (MAV), where OF used with supplemental range meter. For example, in [26] authors consider a Vertical-Take-OFF-and-Landing UAV (VTOL UAV) with an Inertial Measurement Unit (IMU) equipped with suitable filtering algorithms providing reliable estimates of the UAV pose and rotational velocities. Moreover, this UAV equipped with an optoelectronic camera serving as an additional passive sensor whose output is enriching in-flight information. These authors also assume that the UAV control system has an image processing capability to identify a target and to compute OF over the full image basing on well-known Horn and Schunck approach [27].

In [28] a nonlinear controller is presented for a VTOL UAV that exploits a measurement of the average OF to enable hovering and landing onto a moving platform such as the deck of a seagoing vessel.

In a detailed review of copter type UAV’s [11] video control presents a variety of approaches to the usage of natural landmarks and the OF approaches. The principal differences between UAVs of a copter type and even MAV and standard sized UAVs lay in their sizes and velocities. When small-sized UAV is approaching the obstacles with low velocity, the latter can be controlled by the OF signal, since the divergence of the OF field serves as a measure of approaching speed [29,30,31,32]. On the contrary, the standard sized UAVs are landing onto the runway and approaching to the earth by standard glissade during which the altitude changes from hundreds of meters to zero and velocity reduces from dozens of meters per second to meters per second and finally to zero. Simultaneously the rate of the image motion changes on the same orders which lead to the resolution degradation, so the detection of the natural landmarks like in [11] becomes impossible. An application of the OF techniques which determine the image velocity via analysis of the evolution of local image areas needs the coordination of the resolution level of OES with the current speed. The measure of such coordination is obtainable by comparison of measured and theoretically calculated OF velocities.

Even if there are communications related to successful usage of the OF with the aid of two vertically displaced cameras [33,34], more careful analysis shows that with real images and flights the bias between the estimated and real values of the OF achieves unacceptable values [35].

That is why the approach developed for MAV do not apply to the aeroplane landing where the descent velocity cannot be arbitrary controlled. Moreover, the OES resolution when approaching to the earth changes about a hundred times from the start of a glissade. By this reason, the standard OF evaluation does not work accurately if the OES resolution does not reduce in coordination with the UAV altitude. A changing of the camera focal length is somewhat tricky since it needs an additional controlled optomechanical system. Meanwhile, the resolution can be changed by averaging pixels in coordination with estimated OF velocity obtained from the video sequence and its comparison with the value calculated theoretically from exact OF formulas based on current altitude estimation. The principal aim of this article is to demonstrate this approach via real video sequences obtained in real flights. There are two ways to use video navigation in autonomous UAV flights. The first one is a detection of the terrain objects with known coordinates, a definition of their aspect angles and finally, a determining the current UAV coordinates. We demonstrated this approach in [5]. However, if such objects are absent in the field of view, one can determine the current velocity and coordinates by filtering, and the OF is a good way for that. So the combination of direct measurements and OF provides the continuous tracking of the UAV trajectory. This way is convenient in so-called cruise part of the flight, where the altitude and velocity remain almost constant. The landing is the different issue, since the altitude changes on few orders as well as the scale of the picture, therefore, small details become apparent and affect the accuracy of the determining of the OF [36]. That is why at landing phase one needs to adapt the camera resolution and the frame rate following the current altitude. Creation of such cameras is a separate problem, though one can change the resolution by averaging the image signal. In this article, we show how to create such averaging algorithm, which together with filtering based estimation guarantees the reliable evaluation of the altitude at the descent from the altitude of 300 m to 5 m.

The structure of the article is as follows. In Section 2, we present the theory of the OF computation. In Section 3, we give the algorithm of the UAV motion estimation with the aid of the OF and Kalman filtering. Section 4 gives the results of the OF obtained during the real UAV flight at the landing phase and presents the approach to the resolution adaptation from current altitude estimation. In Section 5 we give the algorithms of the averaging scale switching based on the comparison of exact OF values (theoretically calculated) and their estimates with the aid of Lucas-Kanade (L–K) algorithm [37]. It shows that the difference between estimated and calculated OF values may serve as a sensor for the control of averaging. The results of numerical tests showing the reliable tracking of the altitude up to the 5m provided. Section 6 is conclusions where we discuss the results.

## 2. OF Computation: Theory

Since the end of the 70s, many mentions of the OF formulas appeared in the literature, see for example [38]. However, these formulas usually relate to the case of fixed camera position and cannot take into account possible inclination of the line of sight which occurs either during the plane turn (azimuth and roll angles) or during the landing-descent (pitch angle). Meanwhile, the case of a camera with a stabilised line of sight is much more complicated. As an example, the copter needs some inclination angle in order to create propulsion in the flight direction. Hence the pitch angle correction is necessary as it presented in the example in [39]. In recent years navigation by computation of the camera path and the distance to obstacles with the aid of field of image motion velocities (i.e., OF) became highly demanded particularly in the area of relatively small and even micro UAV. Video sequences captured by onboard camera give the possibility of the onboard OF calculation with the aid of relatively simple algorithms like Lucas-Kanade [37] and even more sophisticated ones using higher order terrain illuminance approximations [40,41,42].

Theory of the OF computation in general flight conditions is in [43,44,45]. On that basis, the specialised software IMODEL (version 8.2.o, proprietary) developed and various scenarios of the OF usage in the UAV navigation presented. The IMODEL permits to analyse both the *direct* and *inverse* problems of the OF computation. The *direct* problem is the calculation of the image motion at any point of the field of view for the general orientation of the line of sight. The *inverse* one is the estimation of the OF field for given modelled moving landscape registered by the camera virtually. Moreover, in the implementation stage, the calculus precision problem arises. On one side the higher level filtering algorithm described in [45] and the exact parameters for it affect the resulting precision of the estimated parameters of UAV position. On the other side, the precision achieved is the consequence of the chosen low-level method to determine OF.

An example of the software application is the evaluation of the altitude estimation algorithm with the aid of two vertically displaced cameras [33,35] which shows the presence of bias in the altitude estimation.

**Figure 1:**
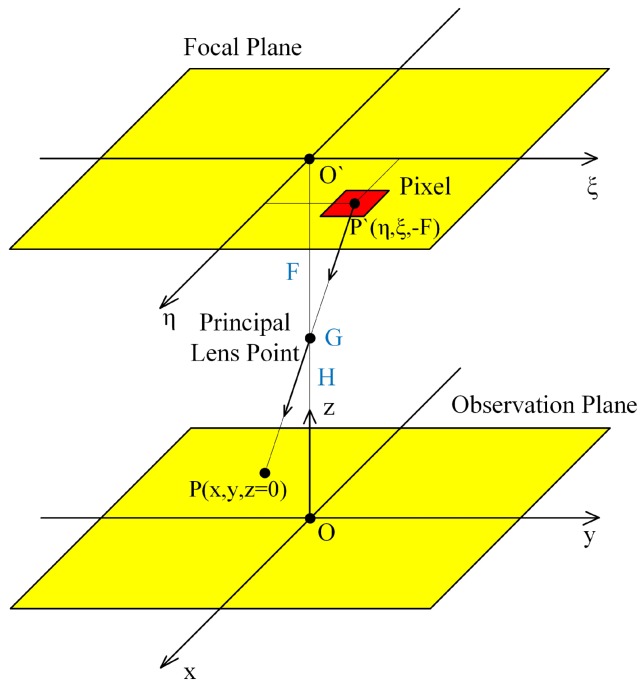
The picture shows the projection of the image plane onto the earth surface. The orientation of the line of sight is changing by rotation at point G.

**Figure 2:**
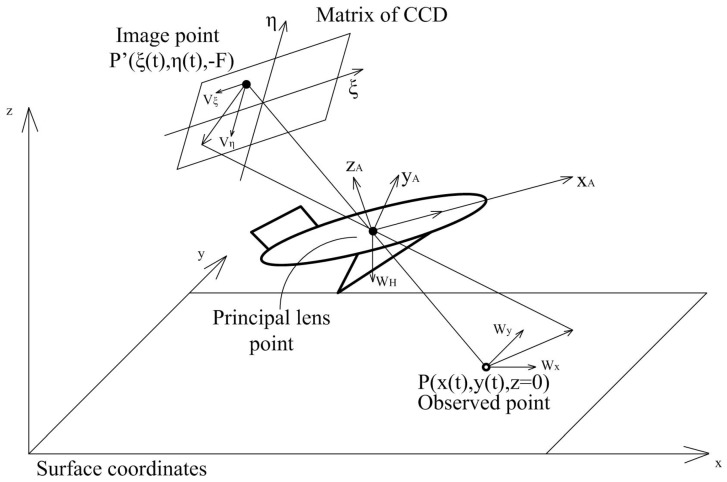
Image motion velocities. **CCD** is the charge coupled devices matrix in the focal plane.

A general approach to the OF computation is based on the pinhole camera geometrical model assuming the flight over a flat surface [39,43,44] (see Figure 1 and Figure 2).

We use the camera coordinate system (ξ,η,−F), where (ξ,η) are the pixel coordinates, ξ corresponds to the direction of flight, η is perpendicular to ξ, and *F* is the lens focal length, and the third coordinate directs to the principal camera point. As it follows from the general theory [16,45] the OF velocities $\left(V_{\xi },V_{\eta }\right)$ equal to

(1) $$\left(\begin{array}{c} V_{\xi }\\ V_{\eta } \end{array}\right)={\left.\left(\begin{array}{c} \frac{\partial \xi }{\partial t}\\ \frac{\partial \eta }{\partial t} \end{array}\right)\right|}_{x=x(\xi ,\eta ,\Lambda (t));y=y(\xi ,\eta ,\Lambda (t))},$$

where dependence on Λ(t) includes the current attitude of the vehicle and orientation of the line of sight. For in-flight computations, one needs to use more extended formula including $W_{x},W_{y},W_{H},\omega_{p},\omega_{r},\omega_{y}$ which is the following matrix equation:

(2) $$\begin{array}{c} \left(\begin{array}{c} V_{\xi }\\ V_{\eta } \end{array}\right)=D_{1}\left(\xi ,\eta ,\Lambda \left(t\right)\right)\left(\begin{array}{c} W_{x}\\ W_{y}\\ W_{H} \end{array}\right)+D_{2}\left(\xi ,\eta ,\Lambda \left(t\right)\right)\left(\begin{array}{c} \omega_{p}\\ \omega_{r}\\ \omega_{y} \end{array}\right), \end{array}$$

where ${\left(W_{x},W_{y}\right)}^{T}$ are the velocities components of the UAV in the plane parallel to the earth surface, ${\left(\omega_{p},\omega_{r},\omega_{y}\right)}^{T}$ are the rotation velocities relative to the line of sight, and $W_{H}$ is the vertical velocity component of the UAV. Exact formulas may found in [39]. By using the estimations of the left-hand side (LHS) of (2) either via Lucas-Kanade (L–K) [37] algorithm or any other, one gets the additional sensor for the UAV velocities estimation. The principal difficulties are the implicit dependence on Λ(t) including the line of sight orientation, given by angles (p,r,y) conditionally called the *pitch, roll, yaw*, and the current altitude *H*.

Therefore, the observation algorithm requires estimation of all mentioned above parameters by Kalman filtering via observation of their derivatives from (2).

The system (2) contains observable variables in the LHS and a set of six variables to be estimated in the right-hand side (RHS). For a sufficiently large field of view, one can evaluate velocities in a vast amount of pixels, while the values are the same for any point. It gives the possibility to solve the issue for example via least square method since the observation model is linear in velocities, and it is possible to estimate unobservable variables via Kalman filtering [45].

**Remark** **1.** *In our approach, we use OF just as an additional sensor to estimate the altitude only. The reason is that in these experiments we use the data obtained with the aid of a series of landing approaches made by a qualified pilot of the light aeroplane. Other parameters such as velocity and orientation angles can be obtained from inertial navigation system (INS) with sufficient accuracy, so we use their nominal values with small corrections made with the aid of Kalman filter (see below (5), (6)). The current value of the altitude is necessary for estimation via OF. In the real flight of the UAV all possible navigation sensors must be used in the fusion with the OF. Here we just test the ability of the OF solely, therefore the real approaches to the runway have been performed by a pilot of a light aeroplane with recording of the video images synchronized with telemetry data from INS and satellite navigation system (SNS).*

## 3. Estimation of the UAV Motion by the OF and the Kalman Filtering

The model of UAV motion described below. It includes the UAV dynamic model and generic measurements model which based on the OF estimation of the UAV attitude velocities.

### 3.1. The UAV Linear Velocity Estimation

The UAV velocity vector $V=col(V_{x},V_{y},V_{z})$ by coordinates x,y,z:

(3) $$\begin{array}{c} V\left(t_{k+1}\right)=V\left(t_{k}\right)+a\left(t_{k}\right)\Delta t+W\left(t_{k}\right), \end{array}$$

where $t_{k}$ is the current time, $t_{k}=t_{0}+k\Delta t$,

$a\left(t_{k}\right)=col\left(a_{x},a_{y},a_{z}\right)$—the vector of accelerations coming from INS (inertial navigation system),
$W(t_{k})$—is the vector of the current perturbations in UAV motion.
  We assume that the components of the perturbation vector are white noises with variances $(\sigma_{x}^{2},\sigma_{y}^{2},\sigma_{z}^{2}).$

Velocity measurements using OF have the following general form:

(4) $$\begin{array}{c} m_{V}\left(t_{k}\right)=V\left(t_{k}\right)+W_{V}\left(t_{k}\right), \end{array}$$

where $W_{V}\left(t_{k}\right)$—are uncorrelated white noises with variances $(\sigma_{V_{x}}^{2},\sigma_{V_{y}}^{2},\sigma_{V_{z}}^{2}).$

Consider relations (3) and (4) for the velocity along *x* axis:

$$\begin{array}{c} V_{x}\left(t_{k+1}\right)=V_{x}\left(t_{k}\right)+a_{x}\left(t_{k}\right)\Delta t+W_{x}\left(t_{k}\right),\\ m_{V_{x}}\left(t_{k}\right)=V_{x}\left(t_{k}\right)+W_{V_{x}}\left(t_{k}\right). \end{array}$$

Velocity along *x* axis estimation on the k+1 step:

$$\begin{array}{c} {\hat{V}}_{x}\left(t_{k+1}\right)=K_{x}\left(t_{k+1}\right)m_{V_{x}}\left(t_{k+1}\right)+\left(1-K_{x}\left(t_{k+1}\right)\right){\tilde{V}}_{x}\left(t_{k+1}\right),\\ {\tilde{V}}_{x}\left(t_{k+1}\right)={\hat{V}}_{x}\left(t_{k}\right)+a_{x}\left(t_{k}\right)\Delta t. \end{array}$$

Kalman filter gives the estimation of ${\hat{V}}_{x}$:

(5) $$\begin{array}{c} {\hat{V}}_{x}\left(t_{k+1}\right)=K_{x}\left(t_{k+1}\right)m_{V_{x}}\left(t_{k+1}\right)+\left(1-K_{x}\left(t_{k+1}\right)\right)\left({\hat{V}}_{x}\left(t_{k}\right)+a_{x}\left(t_{k}\right)\Delta t\right),\\ K_{x}\left(t_{k+1}\right)=\frac{{\hat{P}}^{V_{x}V_{x}}\left(t_{k}\right)+\sigma_{x}^{2}}{{\hat{P}}^{V_{x}V_{x}}\left(t_{k}\right)+\sigma_{x}^{2}+\sigma_{V_{x}}^{2}},\\ {\hat{P}}^{V_{x}V_{x}}\left(t_{k+1}\right)=\frac{\sigma_{V_{x}}^{2}\left({\hat{P}}^{V_{x}V_{x}}\left(t_{k}\right)+\sigma_{x}^{2}\right)}{{\hat{P}}^{V_{x}V_{x}}\left(t_{k}\right)+\sigma_{x}^{2}+\sigma_{V_{x}}^{2}}. \end{array}$$

The formulae for ${\hat{V}}_{y}$ and ${\hat{V}}_{z}$ are analogous.

**Remark** **2.** *Kalman filter coefficients have been derived from empirically registered standard deviations of noise processes. They assumed to be constant during the whole duration of landing. Only the scale rate changes, but after application of the least squares method for estimation of velocities in the RHS of (1) for the large number of observations the remaining noise in observation of the LHS of (1) could be assumed constant for various averaging scales.*

### 3.2. The UAV Angles and Angular Velocities Estimation

UAV angular position estimation is given by three angles $\theta \left(t_{k}\right),\varphi \left(t_{k}\right),\gamma \left(t_{k}\right)$ that are (pitch, roll and yaw, respectively), angular velocities $\omega_{p}\left(t_{k}\right),\omega_{r}\left(t_{k}\right),\omega_{y}\left(t_{k}\right)$ and angular accelerations $a_{p}\left(t_{k}\right),a_{r}\left(t_{k}\right),a_{y}\left(t_{k}\right).$

Pitch angle and pitch angular velocity dynamics described by the following relations:

$$\begin{array}{c} \theta \left(t_{k+1}\right)=\theta \left(t_{k}\right)+\omega_{p}\left(t_{k}\right)\Delta t+a_{p}\left(t_{k}\right)\frac{\Delta t^{2}}{2},\\ \omega_{p}\left(t_{k+1}\right)=\omega_{p}\left(t_{k}\right)+a_{p}\left(t_{k}\right)\Delta t+W_{p}\left(t_{k}\right). \end{array}$$

where $W_{p}\left(t_{k}\right)$—is the white noise with variance $\sigma_{p}^{2}.$

The pitch angular velocity measurement using the OF has the following form:

$$\begin{array}{c} m_{p}\left(t_{k}\right)=\omega_{p}\left(t_{k}\right)+W_{\omega_{p}}\left(t_{k}\right), \end{array}$$

where $W_{\omega_{p}}\left(t_{k}\right)$—is the noise in the angular velocity measurements using OF, which is the white noise with variance $\sigma_{\omega_{p}}^{2}.$

Similarly to the linear velocity estimation we get the pitch angle $\theta (t_{k})$ and pitch angular velocity $\omega_{p}\left(t_{k}\right)$ estimations:

(6) $$\begin{array}{c} \hat{\theta }\left(t_{k+1}\right)=\hat{\theta }\left(t_{k}\right)+{\hat{\omega }}_{p}\left(t_{k}\right)\Delta t+a_{p}\left(t_{k}\right)\frac{\Delta t^{2}}{2}\\ {\hat{\omega }}_{p}\left(t_{k+1}\right)=K_{p}\left(t_{k+1}\right)m_{p}\left(t_{k+1}\right)+\left(1-K_{p}\left(t_{k+1}\right)\right)\left({\hat{\omega }}_{p}\left(t_{k}\right)+a_{p}\left(t_{k}\right)\Delta t\right),\\ K_{p}\left(t_{k+1}\right)=\frac{{\hat{P}}^{\omega_{p}\omega_{p}}\left(t_{k}\right)+\sigma_{p}^{2}}{{\hat{P}}^{\omega_{p}\omega_{p}}\left(t_{k}\right)+\sigma_{p}^{2}+\sigma_{\omega_{p}}^{2}},\\ {\hat{P}}^{\omega_{p}\omega_{p}}\left(t_{k+1}\right)=\frac{\sigma_{\omega_{p}}^{2}\left({\hat{P}}^{\omega_{p}\omega_{p}}\left(t_{k}\right)+\sigma_{p}^{2}\right)}{{\hat{P}}^{\omega_{p}\omega_{p}}\left(t_{k}\right)+\sigma_{p}^{2}+\sigma_{\omega_{p}}^{2}}. \end{array}$$

The formulae for $\hat{\varphi },\hat{\gamma }$ and $\hat{\omega_{r}},\hat{\omega_{y}}$ are analogous.

### 3.3. Joint Estimation of the UAV Attitude

Jointly (5) and (6) give the estimation $\hat{V}$ of the attitude parameters vector, namely:

$$V=col(V_{x},V_{y},V_{z},\omega_{p},\omega_{r},\omega_{y}).$$

This vector measured via OF for each pixel in each frame, which gives according to (2) the overdetermined system of linear equation for V entries.

Since all noises are uncorrelated the covariance matrix *P* for V is diagonal with entries (5), (6).

Integration of velocities V gives the estimation of the UAV position which used for estimation of matrices $D_{1},D_{2}$ in (2).

### 3.4. Discussion of the Algorithm

Many uncertainties remain in the algorithm described above. They may be evaluated only in the real flight with real data. The principal uncertainty is the noise level in formulas (2) and its dependence on the altitude of flight. Experiments show that when approaching to the earth, the tiny details become very important and corrupt the OF estimation. In order to suppress these details, one needs to change resolution but to keep velocity estimation to be correct. In the real flight by averaging the pixel samples one can change resolution, but it is necessary to do it in coordination with the current altitude. Our experiments show that it is possible to change the resolution by comparison of the L–K OF estimation and theoretically calculated OF velocity through the current estimation of the altitude. That is a heuristic approach, and only experiments with real data may show either is it appropriate or not. The difference between two ways of the OF calculation is that L–K does not use the data from INS and even the estimation of the altitude obtained on previous steps with the aid of Kalman filtering. On the other hand, the OF calculation from theoretical model uses the current UAV attitude estimation. In the next section, we demonstrate an approach to the development of such an algorithm to control the scaling.

## 4. OF Estimation in the Real Flight

### 4.1. Examples of the OF Estimation in Approaching to the Earth

**Figure 3:**
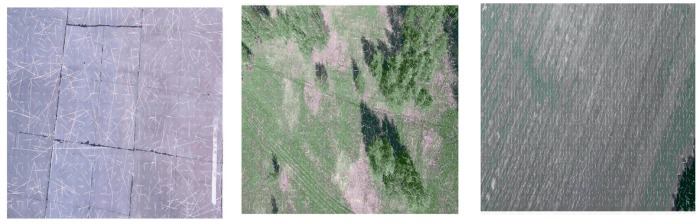
Optical flow estimated at landing with averaging 2×2 at the altitudes 195 m (**right**), 50 m (**centre**), and 20 m (**left**).

**Figure 4:**
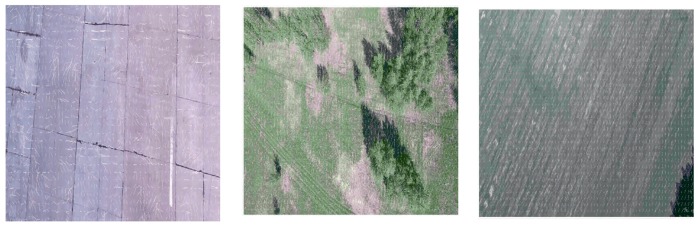
Optical flow estimated at landing with averaging 8×8 at the altitudes 195 m (**right**), 50 m (**centre**), and 20 m (**left**).

In our experiments, we used standard HERO 5 camera (GoPro Inc., San Mateo, CA, USA) and did not change its parameters during the flight. Since the aim of our research is the analysis of possible applications of video navigation in UAV, the camera is at the nose part of the light aeroplane. This aeroplane performed the series of approaches to the runway with accurate recording of the images and telemetric data. During the descent, the resolution of the camera sharpens, and it captures more and more tiny details which prevent good estimation of the image movement. Thereby, the estimation of the OF, which behaves more or less regularly at relatively high altitude, becomes absolutely chaotic when it approaches the earth surface. It is possible to observe this effect in the sequence of frames where the image motion velocities calculation done L–K estimates (see Figure 3), where the image is in almost natural scale with averaging 2×2. It is necessary to coordinate the camera resolution with the current altitude of flight to avoid this effect. Another effect is the increasing image velocity and therefore the additional blurring of the image, which needs the coordinated increasing of the frame rate. Of course, all such features usually do not present in standard cameras and their usage in navigation demands the developments of special observation tools. However, some issues may solve by onboard image processing. It means the artificial change of the resolution by averaging. The effect of the averaging is in Figure 3 and Figure 4.

One can see the estimation of the OF becomes more and more regular with increasing the averaging scale, especially at low altitudes though it is impossible to use the high averaging scale for all altitudes of flight. At low altitudes, the blurring due to the image shift really needs the increasing of the frame rate. At high altitudes, where the averaging leads to the decreasing of the resolution, one cannot get a good quality of the OF estimation.

### 4.2. Switching of Scaling by Means of the Altitude Estimation

**Figure 5:**
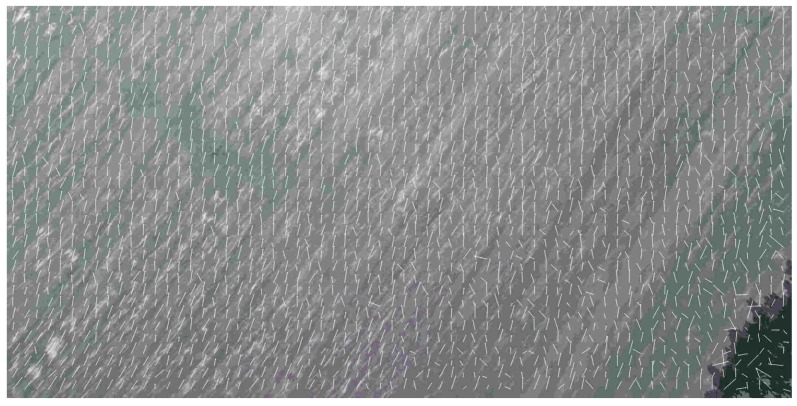
Optical flow estimated by Lucas-Kanade algorithm at the beginning of glissade, height≈ 200 m, level of averaging 2×2. One can observe a rather regular nature of the optical flow which permits to estimate the flight parameters of the unmanned aerial vehicle with more or less high accuracy.

**Figure 6:**
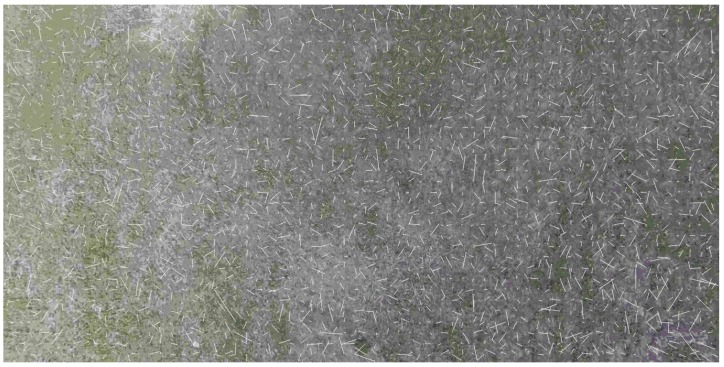
Optical flow estimated by Lucas-Kanade algorithm at the end of glissade, height≈ 30 m, level of averaging 2×2. One can observe the very chaotic nature of the optical flow which prevents the estimation of the flight parameters.

**Figure 7:**
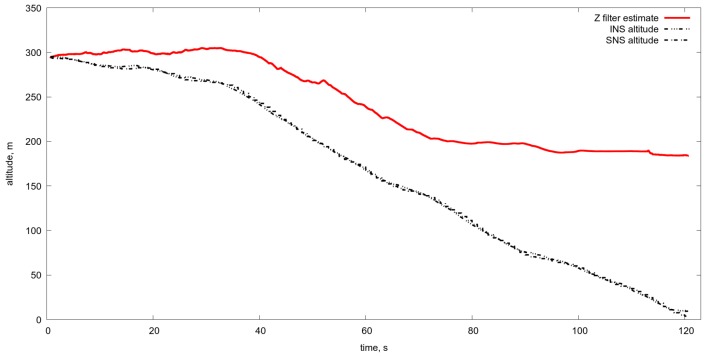
Estimated altitude with the aid of images registered by optoelectronic system without averaging in comparison with the real one given by inertial navigation system with satellite measurements. One can see that estimation on the basis of the Lucas-Kanade algorithm without averaging does not work in the entire range of the altitudes less than 300 m.

**Figure 8:**
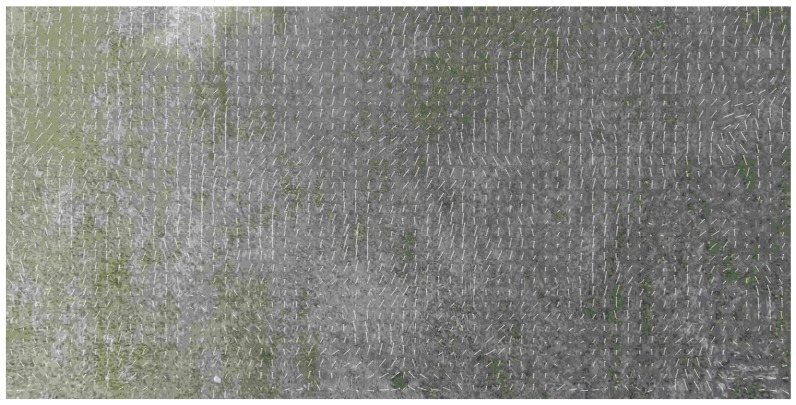
Optical flow estimated by Lucas-Kanade algorithm at the end of glissade, height≈ 30 m, level of averaging 16×16. By comparing with Figure 6 one can observe much more regular behaviour of the optical flow which gives a better estimation of the flight parameters.

**Figure 9:**
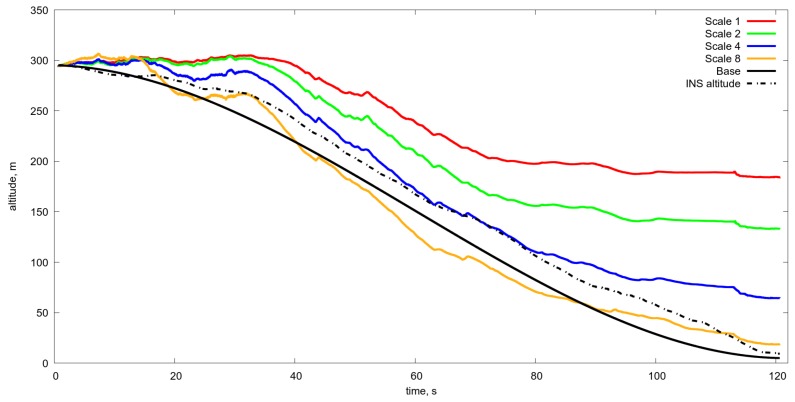
Estimated altitude in comparison with the real one obtained with a various level of resolution with averaging from 1×1 to 8×8. One can see that only the averaging 8×8 gives the acceptable accuracy of the altitude estimation in the range from 300 m to 50 m. Scale 1, 2, 4, 8 estimated altitude obtained by optical flow and Kalman filtering with different level of averaging, namely from 1 to 8. Base is the program motion. INS is the altitude from the inertial navigation system.

**Figure 10:**
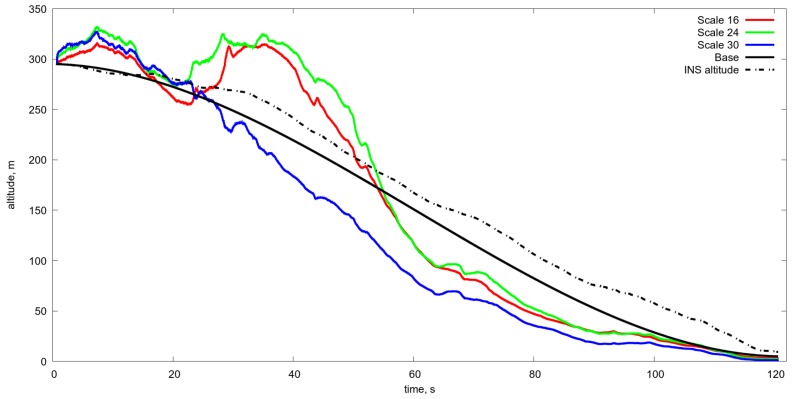
Estimated altitude in comparison with the real one obtained with a various level of resolution with averaging from 16×16 to 30×30. One can see that the averaging greater than 16×16 gives the acceptable accuracy of the altitude estimation in the range from 50 m to 5 m. Scale 16, 24, 30 estimated altitude obtained by optical flow and Kalman filtering with different level of averaging, namely from 16 to 30. Base is the program motion. INS is the altitude from the inertial navigation system.

Another difficulty with the use of the OF is the change of scale and thereby the image shift rate caused by the change of UAV velocity and the flight altitude. Just as an example one can see the estimate of the OF based on Lucas-Kanade [37] algorithm obtained during the real flight at landing from 300 m to the runway (see Figure 5 and Figure 6). As a result, the estimate of altitude, based on the OF estimated by the image of natural scale works more or less satisfactory up to the height 150 m and gives absolutely wrong values after (see Figure 7). Therefore, one needs to manipulate the zoom of the camera (change of focal length) in order to coordinate it with the characteristics of the OF. Meanwhile, one can use another approach, and to change the OES resolution by pixels averaging. The averaging of pixels made the OF calculation somewhat regular even at low altitudes (see Figure 8) with averaging by 16×16 at the altitude ≈30 m. One can compare it with Figure 6 It provides the reliable work of the algorithm up to the height of approximately 5–10 m (see Figure 9 and Figure 10).

In recent work [46], we realised the idea of the scaling control using the current estimation of the altitude. Generally, it is difficult to evaluate the effect of this approach since the estimate of altitude without the use of other sensors is based just on the OF estimation which depends on the altitude itself.

The series of experiments based on the real data shows that it is possible to adapt the level of the averaging in order to expand the range of altitude estimation by the OF.

### 4.3. Test of the Algorithm Based on the Scale Switching via Current Altitude Estimation

As follows from the above consideration one needs to change the resolution of OES in coordination with the height of flight, and of course, the observed image is to be taken into account. Generally it is a nontrivial problem which needs consideration shortly, however now one can suggest the empirical algorithm for the averaging scale changing, based on the data which we have at hands. This empirical algorithm works using current height estimation as described in Table 1 [46].

**Figure 11:**
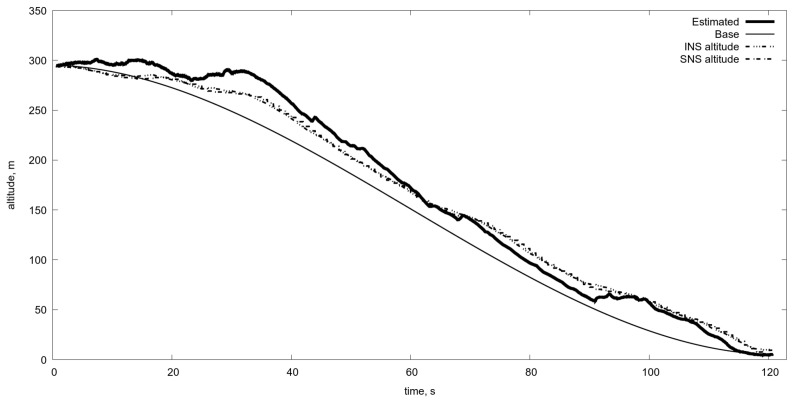
Estimated altitude in comparison with the real one obtained with the aid of averaging algorithm parameters described in Table 1. Estimated is the altitude obtained by optical flow and Kalman filtering. Base is the program motion. INS is the altitude from the inertial navigation system. SNS is the altitude from the satellite navigation system.

One can see that the switching of scale improves the altitude estimation and permits to extend the algorithm operating range (see Figure 11).

Therefore, the possible solution of the OF usage is the changing of the averaging level during the descent. However, what kind of measurements could serve as a sensor for such switching of averaging? In the previous work [46], we examined the switching utilising the altitude estimation. However, at low altitudes all measurements and estimates become unreliable, so we tried to compare the OF velocities computed by L–K algorithm with the OF velocities calculated with the exact formulas and current estimation of the altitude. The parameters of the scaling change algorithm are in Table 1 [46]. The corresponding result of the altitude estimation is in Figure 11. One can observe satisfactory work of the OF in the altitude estimation. However, it is not a clean experiment since the estimation is done using the knowledge of the current altitude which is necessary to transform the OF data into real velocities.

## 5. Scale Switching by the Comparison of Calculated and Estimated OF

### 5.1. Comparison of Calculated and Estimated OF

Here we use another sensor for the scaling switching that is the difference between the estimated and the calculated OF velocities.

**Figure 12:**
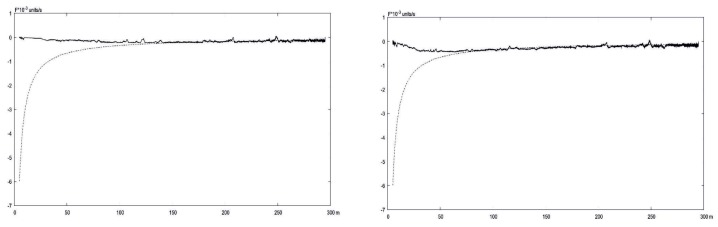
Optical flow velocities via Lucas-Kanade (bold) in comparison with exact values (dots) calculated for different level of scaling: **left**– 4×4, **right**–8×8.

**Figure 13:**
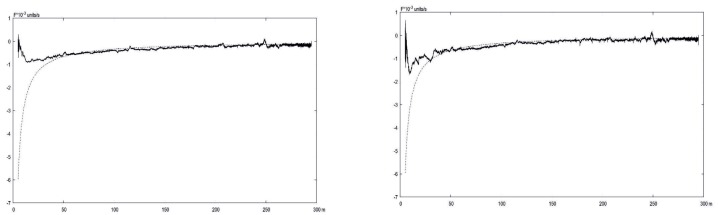
Optical flow velocities via Lucas-Kanade (bold) in comparison with exact values (dots) calculated for different level of scaling: **left**–16×16, **right**–30×30.

The results of experiments related to the comparison of exact and L–K estimated velocities are in Figure 12 and Figure 13. One can observe that the OF becomes useless when close to the earth, though the coordinated increasing of scaling level enlarges the range of reliable measurements.

**Remark** **3.** *In all these pictures and below velocity is measured in $F*10^{-3}units/s,$ where F is the lens focal length in meters.*

In Figure 12 and Figure 13, the exact OF value calculated using (1) with known values of the current flight parameters obtained from INS and corrected with the aid of Kalman filtering. The L–K parameters estimated on from the current video sequence registered by the onboard camera.

### 5.2. OF Estimation as a Sensor for Scaling Switch

**Figure 14:**
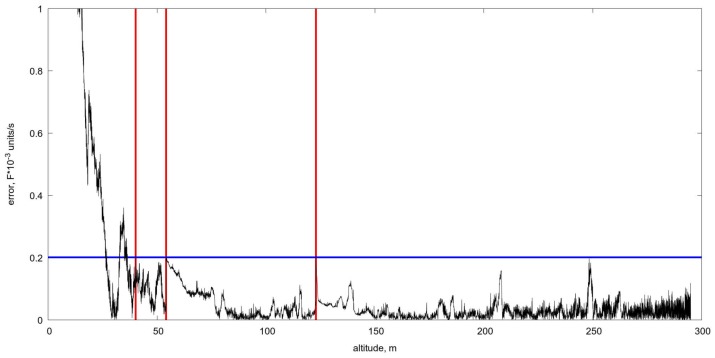
The difference (error) of the optical flow velocities via Lucas-Kanade and exact values, calculated at different altitudes from 300 m to 0 m. Vertical red lines show moments of switching of the averaging levels, that are: 4×4, 8×8, 16×16, and 30×30 from right to left.

**Figure 15:**
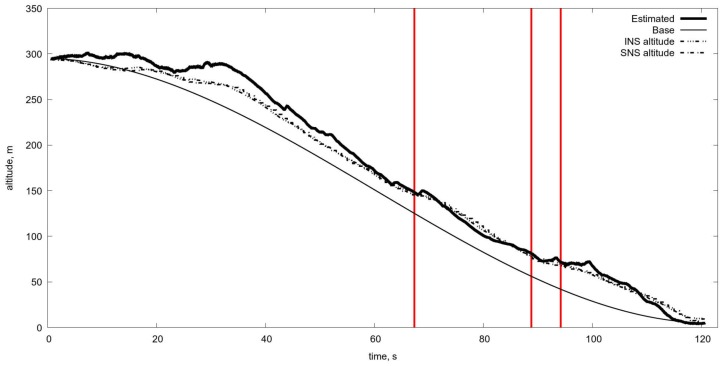
The altitude estimation via optical flow with switching averaging scale. The estimation of the altitude is made on the basis of standard Kalman filtering by fusion of data from the control system and the optical flow measurements. Of course, at low altitudes, less than 25–30 m, the OF measurements are corrupted by very high noise, but the usage of dynamical model and data from control system permit estimate reliably the altitude up to 5 m. Estimated is the altitude obtained by OF and Kalman filtering. Base is the program motion. INS is the altitude from the inertial navigation system. SNS is the altitude from the satellite navigation system.

As specified in the Introduction, we used the series of video sequences captured during the series of descents, where we estimated the OF velocities with the aid of L–K algorithm and compared it with theoretical values corresponding to the current aeroplane altitude. It is evident that the difference increases in approaching to the earth surface, meanwhile the OF permits to evaluate velocity at 25–30 m of height, though the difference becomes unacceptable below. It means that the noise in the velocities estimation via OF exposed to considerable perturbations which corrected by filtering utilising the dynamical model. The usage of Kalman filter based on the UAV dynamical model, observations of the OF, and the control accelerations permit to estimate the altitude in the range from 30–5 m. The picture in Figure 14 shows how to coordinate the scaling with the chosen threshold, which is equal to 0.2 unit/s, starting from averaging 4 × 4 at the beginning of glissade and increasing up to 30 ×30 when the aeroplane is close to the earth, approximately at the height of 5 m. Red vertical lines show the switch of scaling based on the difference of estimated and calculated OF velocities. Figure 15 shows the altitude estimate based only on the OF and filtering. Of course, it is just an experiment demonstrating the ability of the OF in such a complicated situation. In reality, it must combine with other altimeters, but if they fail to work accurately due to some reasons, the OF could serve as a reserved one.

Finally, the comparison of the altitude descent curves can be carried out. The program trajectory is the reference to the other ones. The results of the altitude tracking error are in Table 2. The rightmost column represents overall statistics for the new scale switching algorithm. The scale switch occurs when OF velocity error raises above level 0.2 in Figure 14. Conducted trial shows that the altitude estimate through the video sequence is quantitatively comparable with two other ones.

**Remark** **4.** *In these experiments, the estimation with the aid of L–K algorithm done by standard software and needs the frames from the video sequence and the current scale size in pixels for calculation of the OF velocities. The OF exact value was calculated with the aid of (2) with values of flight parameters obtained from INS corrected by Kalman filter (5),(6). One can observe that the difference of exact and estimated OF velocities gives threshold points which are different in comparison with Table 1.*

## 6. Conclusions

In summary, the article presents the investigation of the OF usage as a sensor at the UAV landing. In general, it needs the adaptation of shooting parameters to the current altitude and velocity of flight that leads to the necessity of changing the camera characteristics such as virtual resolution and the frame rate. However, one can resolve the problem by using image processing such as change the resolution by controllable scaling. By using the difference of the OF velocity estimated by L–K algorithm and calculated via exact formulas and filtered by Kalman estimate as a sensor of the scale switching, it becomes possible to achieve the reliable altitude estimation up to 5 m. It shows how to provide the data fusion of the OF and filtering in the complicated problem of the UAV landing.

## Figures and Tables

**Table 1:** Change of the averaging level as a function of the height estimate.

Height	Scale	Height of Switch
300–50 m	4×4	150 m
150–80 m	8×8	80 m
80–50 m	16×16	50 m
50–5 m	24×24	5 m

**Table 2:** Sample statistics of the altitude tracking error to the program trajectory.

	INS	SNS	Video
Mean, m	17.40	17.43	23.32
Median, m	19.39	19.75	23.85
Minimum, m	−3.36	−4.41	−1.32
Maximum, m	30.04	31.78	46.87
Standard deviation	9.33	10.02	11.60
Standard error	0.12	0.13	0.15
Final error, m	4.42	−1.21	−0.15

## Abbreviations

The following abbreviations are used in this manuscript:

UAV	Unmanned Aerial Vehicle
INS	Inertial navigation system
SNS	Satellite navigation system
OF	Optical flow
LHS	Left hand side
RHS	Right hand side
L–K	Lucas–Kanade (algorithm)
OES	Optoelectronic system
